# Association between 24-h activity patterns and inhibitory control in community-dwelling older adults: a cross-sectional study based on chronic disease status

**DOI:** 10.3389/fpubh.2026.1780172

**Published:** 2026-04-16

**Authors:** Li Zhan, Lin Wang, Shijie Liu, Zhiji Wang, Sijun Wu, Youling Qian, Linxia Tang, Hong Wang

**Affiliations:** 1 School of Physical Education, Wuhan University of Technology, Wuhan, China; 2 Center for Hubei Ethnic Traditional Sports Culture Preservation and Innovation, Wuhan, China; 3 School of Physical Education, Shanghai University of Sport, Shanghai, China; 4 School of Physical Education, Hubei Minzu University, Enshi, China; 5 Department of Physical Education, Shanghai University of Traditional Chinese Medicine, Shanghai, China; 6 School of Wushu, Wuhan Sport University, Wuhan, China

**Keywords:** 24-hour activity patterns, chronic diseases, component data analysis, inhibitory control, older adults

## Abstract

**Objective:**

This study examines the associations of 24-h activity patterns and chronic disease status with inhibitory control in community-dwelling older adults, and as a secondary analysis further explores whether the association differs according to chronic disease status.

**Method:**

A cross-sectional design was employed to facilitate sampling, enrolling 121 community-dwelling older adults aged ≥60 years (75 females; 86 with chronic diseases; mean age 71.4 ± 6.5 years). 24-h activity patterns were recorded with accelerometers, and inhibitory control was measured using the Stroop task. Questionnaires provided information on chronic disease status and other covariates. Component data analysis was applied to explore the relationships between daily activity patterns, chronic disease conditions, and inhibitory control. Results were also examined separately for participants with and without chronic diseases, including group comparisons and proportional substitution analyses.

**Results:**

After adjusting for potential confounders, 24-h activity patterns were significantly related to inhibitory control (*F* = 5.18, *p* = 0.002). Chronic disease status also had a notable main effect (*F* = 5.95, *p* = 0.016), with the chronic disease group scoring 0.43-unit lower in inhibitory control than the non-chronic disease group (*p* = 0.016). In the proportional substitution analysis, shifting 30 min from other activities to moderate-to-vigorous-intensity physical activity (MVPA) resulted in 0.243 increase in inhibitory control (95% CI: 0.02, 0.46). On the other hand, shifting 30 min from MVPA to other activities led to 0.508 decrease in inhibitory control (95% CI: −0.97, −0.04). The interaction effect was not statistically significant. Stratified substitution analyses were treated as exploratory. Although the direction of the associations was similar in both groups, this pattern alone does not support a conclusion that the groups differed. The sensitivity analysis revealed a clear negative relationship between the number of chronic diseases and inhibitory control: each additional chronic disease was linked to an average 0.178-unit decrease in inhibitory control (95% CI: −0.28,−0.07; *p* = 0.0009), consistent with the main results.

**Conclusion:**

In older adults' 24-h activity patterns, a greater relative share of MVPA was associated with better inhibitory control, whereas having chronic diseases was independently related to poorer inhibitory control. No significant interaction was found, and the stratified results should be viewed with caution and validated in larger studies with longer follow-up.

## 1 Introduction

As populations around the world age rapidly, the number of people with dementia is steadily growing, with estimates rising from 57.4 million in 2019 to 152.8 million by 2050 (1). This growing burden highlights the need to prioritize cognitive health in later life and strengthen efforts to prevent dementia. Inhibitory control is an important aspect of executive function. It refers to the ability to intentionally block out irrelevant or unsuitable thoughts, emotions, and behaviors to stay focused on a goal (2–4). For older adults, intact inhibitory control provides an important cognitive basis for independent and safe daily living (5). Evidence indicates that inhibitory control, which is largely supported by the prefrontal cortex, is especially vulnerable to age-related changes and commonly shows a gradual decline with advancing age (4). This decline can negatively affect older adults' ability to adapt to their surroundings, make sound decisions, and maintain a good quality of life (6, 7). Adding to the complexity, this age-related decline often occurs alongside a growing burden of chronic disease. For instance, in 2018, diabetes prevalence among Chinese adults was 11.1% in the 40–49 age group, rising to 23.9% among those aged 60–69 and 27.3% among those aged 70 and above, which is substantially higher than in middle-aged adults (8). This tight connection between population aging and chronic disease increases health risks in later life (9). Many studies suggest that chronic conditions can trigger several harmful biological processes, including persistent low-grade inflammation (10, 11), oxidative stress (12), vascular endothelial dysfunction (13, 14), and metabolic disruption (15, 16). Over time, these processes may gradually damage key brain regions, including the prefrontal cortex, and in turn impair inhibitory control in older adults. Against the combined challenges of rapid population aging and a growing burden of chronic disease, it is important to identify and test effective strategies that can slow declines in inhibitory control, with clear public health relevance.

Daily behaviors are constrained within a fixed 24-h (1,440-min) day. Sleep, sedentary behavior, and physical activity operate in a time-use trade-off system, such that increasing the duration of one behavior inevitably reduces the time allocated to others (17). Accordingly, these behaviors should be considered as parts of a full 24-h composition rather than examined in isolation. In addition, time-use data are closed and co-dependent, meaning that more time spent in one behavior necessarily corresponds to less time spent in others. This property can lead to spurious correlations and multicollinearity when conventional regression models are applied to raw time-use variables. Compositional data analysis (CoDA) is particularly appropriate for activity patterns research because it treats behaviors as mutually dependent parts of a whole and allows estimation of both overall associations and the modeled effects of reallocating time between behaviors (17–19). Recent reviews have shown that CoDA-based reallocation research has expanded rapidly, while also underscoring the importance of clear reporting and careful interpretation in observational studies (19, 20).

Within this framework, a growing body of research has linked 24-h activity patterns to cognitive and health-related outcomes in older adults. Recent studies have further advanced this integrated perspective in older adults (21–24). Evidence from Chinese older populations indicates that reallocating sedentary time to physical activity and sleep is associated with better physical and mental health. In addition, although overall adherence to the 24-h movement guidelines remains low, compliance has been significantly associated with a range of health outcomes (25, 26). Existing research further suggests that physical activity may help slow declines in inhibitory control in older adults through several pathways, such as improving cerebral blood flow, supporting neuroplasticity, and reducing inflammation (27–29). The benefits appear to be especially strong for moderate-to-vigorous-intensity physical activity (MVPA), defined as activity of at least moderate intensity (≥3 METs) (30). In contrast, greater sedentary time and shorter sleep duration, as well as poorer sleep quality, have been associated with worse inhibitory control, potentially due to reduced cerebrovascular stimulation and impaired arousal regulation (31–33).

However, evidence specifically linking 24-h activity patterns to cognitive outcomes in older adults remains relatively limited. In particular, most studies have treated older adults as a relatively homogeneous group and rarely examined whether these associations differ by health status, despite likely heterogeneity (21, 23). Moreover, although some studies have focused on older adults with chronic diseases, there is still limited direct evidence on whether chronic disease status modifies the association between 24-h activity patterns and inhibitory control in broader community-dwelling older populations (34). Therefore, using a CoDA framework, the present study primarily examined the association between 24-h activity patterns and inhibitory control in community-dwelling older adults, as well as the independent association of chronic disease status with inhibitory control. As a secondary analysis, we further explored whether the association between 24-h activity patterns and inhibitory control differed according to chronic disease status. To broaden evidence beyond chronic-disease-only samples, we included a disease-free comparison group and evaluated potential effect modification by chronic disease status/burden within a community-based sample.

## 2 Materials and methods

The reporting of this cross-sectional study followed the STROBE guidelines, and the completed checklist is provided in Supplementary Table 1.

### 2.1 Participants

Using GPowerWin_3.1.9.7, an *a priori* sample size calculation was performed for the primary regression model. Assuming a medium effect size (*f*^2^ = 0.15), a significance level of α = 0.05, and 80% power, the minimum required sample size was estimated to be 114 participants. Using convenience sampling, the study recruited older adults from three university-affiliated residential communities at Wuhan University of Technology, including the East and West residential communities on the Mafangshan Campus and the Youli community. Ultimately, 138 participants were recruited. Inclusion criteria were: (1) age ≥60 years; (2) community-dwelling; (3) able to ambulate independently and perform usual activities of daily living without assistance; (4) able to communicate and understand task instructions and to complete the Stroop practice trials; and (5) willing to participate and provide written informed consent. Exclusion criteria were: (1) self-reported physician diagnosis of dementia or other major neurological disease; (2) hearing, vision, or communication impairments that precluded completion of the Stroop task; (3) severe health conditions that made unsupervised physical activity medically unsafe or substantially limited functional mobility. Data were collected between September 18, 2024, and April 15, 2025. After excluding participants due to insufficient GT3X wear time, incomplete baseline information, or missing Stroop task test data, 121 older adults were ultimately enrolled (46 males and 75 females). The participant recruitment, exclusion, and final inclusion process is shown in Supplementary Figure 1. The data for this study were drawn from the same community-based baseline project reported by Wang et al. (34). This analysis included 121 participants, 75 of whom overlapped with the previous study. Nevertheless, this study focuses on inhibitory control, employs different research questions and analytical frameworks, and includes a sample comprising both older adults with and without chronic diseases. To clearly distinguish between the two studies and ensure transparency, Supplementary Table 2 provides a detailed summary of the key differences between this study and the previously published work regarding participant overlap and study design. The study received approval from the Medical Ethics Committee of Wuhan Sport University (Ethics Approval No.: 2025119). All participants have signed written informed consent forms.

### 2.2 Measures

#### 2.2.1 Assessment of 24-h activity patterns

Participants' 24-h activity patterns were measured using an ActiGraph wGT3X-BT accelerometer. Before data collection, the study purpose, procedures, and key precautions were explained to each participant. Participants were provided with accelerometers and instructed to wear them on their non-dominant wrist for seven consecutive days (including 5 weekdays and 2 weekend days), removing them only for bathing and other water-based activities. The accelerometer was worn on the non-dominant wrist to reduce potential bias from dominant-hand movements, given that dominant vs. non-dominant wrist placement can yield systematic differences in free-living estimates (35). Data were recorded in 60-s epochs. Non-wear periods are defined as continuous 60-min intervals where activity counts fall below the detection threshold. Throughout the monitoring period, researchers checked in daily and reminded participants to keep wearing the device to improve compliance. On the 8 day, the accelerometers were retrieved, and data were downloaded using ActiLife 6.13.3. Following Hart (2011), a day was considered valid if wear time was at least 10 h. Data were included only when participants provided at least 3 valid days (2 weekdays and 1 weekend day) (36). Activity intensity was classified using the count-based cut-points proposed by Freedson: sedentary behavior (SB), 0–99 counts/min; light-intensity physical activity (LPA), 100–1,951 counts/min; and MVPA, ≥1,952 counts/min (37). This study adopted thresholds that have been widely validated and are commonly used in research on physical activity among older adults to ensure the comparability of results. To improve the accuracy of sleep duration classification, participants were provided with paper sleep diaries prior to monitoring. Sleep periods were initially identified based on accelerometer data and then cross-checked against the bedtime and wake-up times recorded in the diaries.

#### 2.2.2 Assessment of inhibitory control

The experimental program was developed using E-prime software and employed an adapted Stroop task paradigm to assess participants' inhibitory control. This task has been validated among older adults (38).Throughout the entire testing process, participants were tested in a quiet room, well-lit indoor environment. Once the experiment begins, the four Chinese characters (“红”, “黄”, “蓝”, “绿”) will appear randomly in the center of the screen, each written in a different color.The experimental tasks are divided into two main categories: congruent and incongruent conditions. A congruent condition occurs when the presented word matches its displayed color, such as when the word “黄” appears and is also colored yellow. An incongruent condition occurs when the presented word does not match its displayed color, such as when the word “黄” appears but is colored green. Trials under both conditions appear randomly and with equal probability. Participants evaluated word meaning and ink color for each stimulus pair and responded via button press. At the beginning of each trial, a “+” fixation point appears at the screen for 500 ms, followed by a random stimulus for 1,500 ms. There is a 500 ms interval between the end of the stimulus and the start of the next trial. The Stroop task consists of a practice phase (16 trials) and a formal phase (72 trials). Before the main experiment, a practice session was held. Afterward, participants received feedback on their response accuracy (AC) and reaction time (RT). The formal task began only after the instructions were clearly understood. No feedback was provided during the formal experiment. Correct response AC and RT were recorded for both congruent and incongruent conditions.

This study used the Balanced Integration Score (BIS) to index inhibitory control in older adults. BIS, proposed by Liesefeld, combines response speed and accuracy using a standardized approach (39). In brief, AC and RT were first calculated separately for the congruent and incongruent conditions. To place both conditions on a common scale, AC and RT values from the two conditions were pooled for z-standardization, and zBIS was then computed for each condition as: zBIS = z (AC) – z(RT). The resulting zBIS values (congruent and incongruent) were rounded to two decimal places. To index inhibitory control, the final Stroop-based zBIS score was calculated as the incongruent-condition zBIS minus the congruent-condition zBIS. Higher zBIS values indicate better inhibitory control (22).

#### 2.2.3 Assessment of chronic disease

A paper-based questionnaire provided participants with a list of chronic diseases based on the International Classification of Diseases, 10th Revision (ICD-10). The list covered seven types of chronic diseases: cardiovascular and cerebrovascular diseases (e.g., heart disease, cerebrovascular disease, hypertension); metabolic diseases (diabetes mellitus, dyslipidemia, etc.); digestive and hepatobiliary diseases (peptic ulcers, cholecystitis, fatty liver, chronic hepatitis, etc.); respiratory chronic diseases (chronic bronchitis, emphysema, etc.); musculoskeletal and bone metabolic diseases (arthritis, osteoporosis, etc.); kidney diseases (chronic nephritis, etc.); neoplasms (cancer or malignant tumors). All disease information was self-reported by participants based on prior physician diagnoses. Participants reporting at least one condition were classified as having chronic disease, whereas those reporting none were classified as disease-free. Accordingly, participants were grouped as 0 conditions vs. one or more conditions (40, 41). For the sensitivity analysis, the total number of reported chronic diseases was summed across conditions and modeled as a continuous variable.

#### 2.2.4 Covariates

Paper-based questionnaires were distributed to collect demographic data from older adults, including age, gender, height, weight, educational attainment, and economic status. Body mass index (BMI) was calculated as weight (kg) divided by height (m) squared. Educational attainment is categorized into primary school and below, secondary school and higher education, or higher levels (41). Economic status is assessed based on the average monthly income of household members and divided into two categories: ≤ 5,000 RMB and >5,000 RMB (42).

### 2.3 Statistical analysis

All statistical analyses were conducted in R 4.5.1 using the *robCompositions* package for component data analysis. Daily activity comprised SB, LPA, sleep and MVPA. Since the sum of time spent in these four behavioral categories is constant at 24 h, the closed-loop nature of component data may lead to spurious correlations and multicollinearity issues in traditional regression analyses. This study employs isometric log-ratio (ILR) transformation to convert 24-h activity patterns data from simplex space into real Euclidean space (43). This approach reduces collinearity arising from fixed effects and constraints, enabling simultaneous analysis of all activity behaviors within a single linear model (23, 24). None of the behavioral variables have zero values, so no imputation is required.

For each participant, the four behaviors were assembled into a four-part vector (Sleep, SB, LPA, MVPA) and rescaled by closure so that the total equaled 1,440 min/day. The resulting composition was then transformed using isometric ILR to obtain three orthonormal coordinates, which were entered together in the regression model to represent the full 24-h composition. To make the results easier to interpret for each behavior, we followed (71) and generated four pivot-coordinate sets, taking Sleep, SB, LPA, and MVPA as the pivot component in turn (17). In each set, the first ILR coordinate contrasts the pivot behavior with the geometric mean of the other three behaviors, so its coefficient indicates whether a higher relative share of that behavior (and a corresponding reduction in the others) is associated with higher or lower zBIS after covariate adjustment. The remaining two coordinates capture the relative balances among the non-pivot behaviors.

A linear regression was applied to evaluate the association of 24-h activity patterns with inhibitory control (44). Given that chronic disease status may introduce heterogeneity and potentially modify the association between the activity composition and inhibitory control, we first estimated the overall main effects and then tested the interaction. Model 1 was specified with zBIS as the outcome, and ilr-transformed 24-h activity compositions as the main predictor. Include covariates such as chronic disease status, age, gender, BMI, education level, and economic status. Employ Type II ANOVA to evaluate the main effects of 24-h activity patterns and chronic disease status separately. Building on this framework, we further fitted Model 2, which included the interaction term. A Type III ANOVA was used to test the joint effect of activity patterns and chronic disease status. The interaction term was not statistically significant. Accordingly, the principal findings of this study are interpreted primarily on the basis of the main effects estimated in Model 1. To further quantify the independent effect of chronic disease status, adjusted marginal mean estimates were calculated for both groups of older adults with and without chronic diseases using the same model. The between-group adjusted mean difference was estimated, along with its 95% confidence interval (*CI*) and *p* value (45, 46).

Considering that “one-to-one” time substitution uses absolute duration as the allocation unit, it may not fully account for the proportion of time each behavior occupies within the 24-h activity patterns. As a result, it may overstate the effects of behaviors with low proportions (such as MVPA) or underestimate the impact of behaviors with high proportions (such as SB or sleep). In contrast, proportional substitution methods such as the “one-to-three” approach allocate time according to the relative duration of each behavior. Because this method reflects how behaviors are distributed across the 24-h activity composition, it may reduce the distortion introduced by simple absolute substitution and provide a clearer estimate of the trade-offs among behaviors. Accordingly, we conducted one-to-three proportional reallocation analyses. The sample geometric mean composition served as the reference. In each scenario, one target behavior (sleep, SB, LPA, or MVPA) was increased or decreased by 30 min/day, while the other three behaviors were adjusted using the same proportional multiplier so that the total remained fixed at 1,440 min/day. Both the reference composition and each reallocated composition were then transformed using the same ILR basis applied in Model 1 and entered into Model 1 to generate the corresponding predicted zBIS values. The effect estimate was defined as the predicted zBIS for the reallocated composition minus the predicted zBIS for the reference composition. Uncertainty was assessed using bootstrap resampling (1,000 resamples) to derive 95% confidence intervals for the predicted difference (47). These findings represent model-based predictions rather than observed changes in accelerometer-derived behaviors.

Additionally, to examine whether chronic disease status influenced the relationship described above, participants were classified as either with chronic disease or without chronic disease within the same compositional regression framework. Changes in zBIS were then estimated under proportional reallocation scenarios of up to 30 min/day, and response curves were plotted to illustrate the predicted changes in inhibitory control for both groups across time reallocations. On these plots, the *x*-axis represents minute increases or decreases relative to the sample mean time, and the *y*-axis represents the corresponding predicted change in inhibitory control. To minimize potential bias from treating chronic disease status as a simple binary variable, chronic disease burden was also modeled as a continuous measure based on a standardized list of conditions. Using the same robust pooled regression framework, a sensitivity analysis was conducted to estimate the regression coefficient for the number of chronic diseases, reflecting the expected change in zBIS associated with each additional chronic disease category.

## 3 Results

### 3.1 Participant characteristics

The analytic sample included 121 eligible older adults with valid accelerometer data, Stroop task results, and complete baseline information. Among them, 46 were men (38.0%) and 75 were women (62.0%), with a mean age of 71.4 ± 6.5 years (Table 1). Overall, 86 participants (71.1%) reported having chronic diseases, whereas 35 (28.9%) did not. When stratified by chronic disease status, the two groups showed generally similar baseline characteristics, including age, sex, education, BMI, socioeconomic status, and key Stroop task measures (*p* > 0.05).

**Table 1:** Baseline characteristics of participants.

Characteristic	Total (*n* = 121)	Participants with chronic diseases (*n* = 86)	Participants without chronic diseases (*n* = 35)	Statistic	*p*
Age, *y* mean (SD)	71.4 (6.5)	72.6 (6.2)	69.89 (7.2)	*t* = −1.570	0.122
Sex, *n* (%)					
Male	46 (38.0)	33 (38.4)	13 (37.1)	*χ^2^* = 0.016	0.899
Female	75 (62.0)	53 (61.6)	22 (62.9)		
Education, *n* (%)					
Primary	11 (9.1)	9 (10.5)	2 (5.7)	*χ^2^*=3.801	0.150
Secondary	54 (44.6)	42 (48.8)	12 (34.3)		
Tertiary	56 (46.3)	35 (40.7)	21(60)		
Ses, *n* (%)					
≤ 5,000	51 (42.1)	37 (43)	14 (40)	*χ^2^* = 0.093	0.760
>5,000	70 (57.9)	49 (57)	21 (60)		
BMI, mean (SD)	23.2 (2.4)	23.5 (2.5)	22.6 (2.3)	*t* = −1.857	0.066
Time-use composition, min/d compositional mean (%)					
Sleep	520.18 (36.12)	498.6 (34.63)	571.1 (39.66)		
SB	714.04 (49.59)	748.7 (51.99)	628.7 (43.66)		
LPA	160.54 (11.15)	153.1 (10.63)	178.4 (12.39)		
MVPA	45.24 (3.14)	39.6 (2.75)	61.8 (4.29)		
Stroop task: mean (SD)					
congruent AC, %	86.96 (12.49)	87.4 (12.5)	85.9 (12.7)	*t* = −0.588	0.588
congruent RT, ms	914.84 (132.77)	914.2 (137.5)	916.4 (122.1)	*t* = 0.080	0.936
Incongruent AC, %	82.39 (13.86)	81.0 (14.5)	85.7 (11.6)	*t* = 1.683	0.095
Incongruent RT, ms	1,101.98 (147.17)	1,113.4 (155.0)	1,074.0 (123.5)	*t* = −1.339	0.183

Based on the geometric mean composition of the 24-h activity patterns, participants spent 520.18 min (36.12%) sleeping, 714.04 min (49.59%) in SB, 160.54 min (11.15%) in LPA, and 45.24 min (3.14%) in MVPA. Descriptively, compared with those without chronic diseases, participants with chronic diseases tended to spend more time SB and less time sleeping and physically active.

### 3.2 Examining whether chronic disease status modifies the association between 24-h activity patterns and inhibitory control in older adults

The results of the Type II ANOVA for Model 1 indicate that, after controlling for confounding factors, older adults' overall 24-h activity patterns were significantly correlated with inhibitory control (*F* = 5.18, *p* = 0.002) (Table 2). This indicates that inhibitory control in older adults is associated with how daily time is allocated across activities. At the same time, the chronic disease status also showed a significant independent main effect on inhibitory control (*F* = 5.95, *p* = 0.016), indicating that, controlling for other factors, older adults with chronic diseases were associated with lower overall inhibitory control levels. The effects of age, gender, BMI, educational attainment, and socioeconomic status did not reach statistical significance (*p* > 0.05).

**Table 2:** Analysis of deviance table (Type II /III tests).

Predictor	Inhibitory control (*n* = 121)		
	***F***	***P***	**Adjusted** ***R**^2^*
Model 1: activity patterns vs. inhibitory control			
Activity patterns	5.18	0.002^*^	0.273
Sex	3.77	0.055	
Age	2.33	0.130	
Education	1.37	0.258	
BMI	1.48	0.226	
ses	0.12	0.732	
Chronic disease	5.95	0.016^*^	
Model 2:model 1+chronic disease interaction with			
activity patterns			
Chronic disease ^*^activity patterns	0.40	0.754	0.26

^*^indicate statistical significance at *p* < 0.05.

Building on this framework, we further fitted Model 2, which included the interaction term. A Type III ANOVA was used to test the joint effect of activity patterns and chronic disease status. The interaction term was not statistically significant (*F* = 0.40, *p* = 0.754), indicating that, in this sample, there was no evidence that chronic disease status meaningfully modified the overall association between 24-h activity patterns and inhibitory control. Given the lack of a significant interaction, the main findings of this study are interpreted primarily on the basis of the main effects from Model 1, which did not include the interaction term.

### 3.3 Linking 24-h activity patterns to inhibitory control in older adults

Given the significant main effect of 24-h activity patterns in Model 1, a proportional substitution analysis was subsequently conducted (Table 3). The linear regression results for each subcomponent of 24-h activity patterns and components of inhibitory control in older adults are detailed in Supplementary Table 3. Importantly, the reallocation results reported below reflect model-based hypothetical substitutions, rather than observed changes in accelerometer-derived activity data. Using the sample geometric mean as a reference, when 30 min were proportionally reallocated to MVPA, the inhibitory control prediction value increased by 0.243 units (95% CI: 0.02, 0.46). Conversely, when 30 min were transferred out of MVPA, the inhibitory control prediction value decreased by 0.508 units (95% CI: −0.97, −0.04). Beyond the two scenarios described above, when redistributing 30 min of time between LPA, sleep or SB and other behaviors, the predicted changes in inhibitory control were close to zero, with 95% CIs spanning zero. Overall, under the premise of an unchanged 24-h total time structure, the relationship between time reallocation between MVPA and other behaviors and inhibitory control levels was the most sensitive. Increasing MVPA may help maintain or improve inhibitory control abilities in older adults.

**Table 3:** Predicted change in inhibitory control associated with reallocating 30 min between 24-h time-use pattern.

Behavior	+30 min reallocated to this behavior	−30 min reallocated from this behavior
Sleep	−0.007 (−0.07, 0.05)	0.007 (−0.05, 0.07)
SB	−0.026 (−0.06, 0.01)	0.026 (−0.01, 0.06)
LPA	−0.014 (−0.11, 0.07)	0.017 (−0.08, 0.13)
MVPA	0.243 (0.02, 0.46)^*^	−0.508 (−0.97, −0.04)^*^

^*^indicate statistical significance at *p* < 0.05.

### 3.4 The impact of chronic disease status on inhibitory control in older adults

Revised based on Model 1, the adjusted marginal means showed poorer inhibitory control in participants with chronic diseases compared with those without. After controlling for age, sex, BMI, education, socioeconomic status, and 24-h activity patterns, the adjusted zBIS was −1.74 in the chronic disease group vs. −1.31 in the non-chronic disease group (Table 4). The adjusted between-group difference was −0.43 (95% CI: −0.786, −0.081; *p* = 0.016). The full adjusted regression coefficients for Model 1 are presented in Supplementary Table 4. Overall, chronic disease status was inversely associated with inhibitory control, indicating weaker inhibitory control performance among older adults with chronic conditions.

**Table 4:** Impact of chronic disease status on inhibitory control.

Chronic disease	Emmean	SE	95% CI
0	−1.31	0.167	(−1.64, −0.976)
1	−1.74	0.119	(−1.97, −1.505)

Estimated marginal means (emmean) of inhibitory control z-scores by chronic disease status. 0 = non-chronic disease, 1 = at least one chronic disease. SE, standard error; CI, confidence interval.

### 3.5 A stratified analysis of the association between 24-h activity patterns based on chronic disease status and inhibitory control in older adults

**Figure 1:**
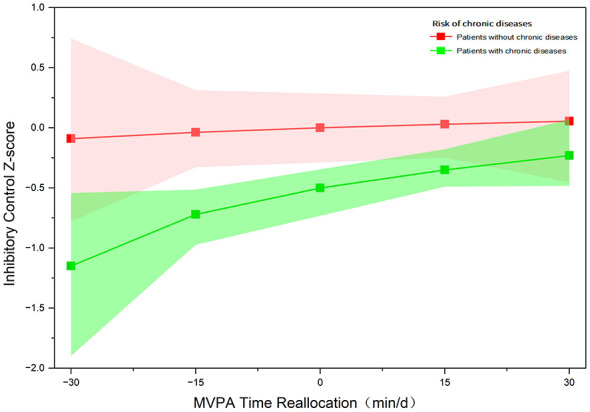
Predicted change in inhibitory control associated with proportional reallocation of MVPA time by chronic disease status. Lines represent model-predicted changes in zBIS when time is reallocated to or from MVPA relative to the sample mean composition. The red line indicates participants without chronic diseases, and the green line indicates participants with chronic diseases. Shaded areas represent 95% CIs.

After adjusting for confounding factors, we further stratified the results of proportional substitution by chronic disease status (Figure 1). In both groups, reallocating time from other behaviors to MVPA was associated with higher predictive inhibitory control, whereas reallocating time from MVPA to other behaviors was associated with lower predictive inhibitory control. However, as the interaction term was not statistically significant, these stratified results should be considered exploratory and descriptive. The point estimates in the chronic disease group appear numerically larger, but the confidence intervals in the non-chronic disease group are wider, which may reflect its smaller sample size and lower precision.

### 3.6 Sensitivity analysis

To test the robustness of the findings, we conducted an additional analysis using the number of chronic diseases as the exposure variable. After adjustment for 24-h activity patterns and relevant covariates, the number of chronic diseases showed a significant negative dose-response association with inhibitory control. Specifically, each additional chronic disease was associated with an average 0.178-unit decrease in zBIS (95% CI: −0.28 to −0.07, *p* = 0.0009) (Supplementary Table 5). This result was consistent with the primary analysis, suggesting that the main conclusions remained stable when the binary indicator of chronic disease status was replaced with the number of chronic diseases.

## 4 Discussion

### 4.1 Sample characteristics and activity patterns

Research indicates that the daily activity time of older adults is primarily distributed between SB and sleep, with SB accounting for 49.59% and MVPA only 3.14%, revealing a pattern of more sitting and less movement. Compared with those without chronic diseases, older adults with chronic conditions spent more time SB, engaged in less physical activity, and slept less overall. This finding aligns with Li et al. (48). The aforementioned phenomenon may be partly related to functional limitations caused by pain or discomfort associated with chronic diseases, thereby reducing physical activity and increasing SB (49). At the same time, depression, anxiety, and concerns about activity exacerbating symptoms may reduce their willingness to engage in physical activity (50, 51). On the other hand, increased awakenings caused by symptoms such as nocturnal discomfort and nocturia, along with the potential impact of psychological burdens and medication factors, may all contribute to reduced sleep duration among chronic disease patients (32, 52).

### 4.2 Independent effects of 24-h activity patterns and chronic disease status on inhibitory control in older adults

After controlling for multiple confounding factors, a significant association was found between 24-h activity patterns and inhibitory control in older adults. This aligns with the findings obtained by Collins et al. using component data analysis, further supporting the present study's discovery regarding the association between 24-h activity patterns and inhibitory control performance (21). In the proportional substitution model of components, allocating more time to MVPA is associated with higher inhibitory control performance, whereas shifting time away from MVPA is linked to reduced inhibitory control levels. In contrast, proportionally redistributing time between sleep, SB, and LPA had a smaller and often non-significant impact on inhibitory control, suggesting that not all components of 24-h activity patterns hold equal importance for inhibitory control. The reason may lie in the fact that adjustments in sleep duration, LPA, and sedentary behavior at the physiological level may not be sufficient to significantly alter the neural and cardiovascular pathways associated with inhibitory control. In contrast, the higher-intensity load represented by MVPA is more likely to trigger substantial cardiovascular and neural responses (53, 54). Therefore, MVPA exhibits a relatively stronger association under scenarios involving time reallocation. Overall, this study suggest that MVPA may be a key behavioral activity for maintaining and enhancing inhibitory control abilities, consistent with previous research based on objective measurements (21, 55).

Controlling for 24-h activity patterns and related confounding factors, this study found that chronic disease status was independently and negatively associated with inhibitory control. Older adults with at least one chronic disease exhibited significantly lower inhibitory control levels, with an average decrease of approximately 0.43 units. In a longitudinal study of community-dwelling older adults, baseline diabetes was significantly associated with a larger decline in Stroop test performance over 2 years (56). This pattern is consistent with our findings and suggests that chronic metabolic conditions such as diabetes, and more broadly a greater chronic disease burden, may be linked to poorer performance on inhibitory control-related tasks.

It is worth noting that chronic diseases in older adults rarely exist in isolation as single conditions or through singular pathways. Instead, they more commonly manifest as multiple coexisting conditions and specific clusters of comorbidities (57). Population cohort studies have also demonstrated that different patterns of multimorbidity and their cumulative processes are associated with distinct trajectories of functional decline (58). Therefore, simplifying chronic diseases into a binary classification of presence or absence may fail to adequately reflect the gradient of cumulative disease burden, thereby limiting the identification of risk gradients and dose-response relationships. As a robustness check, chronic disease burden was also modeled as a continuous index based on the same condition list, and sensitivity analyses were performed. Within a regression framework adjusting for 24-h activity composition and relevant covariates, a clear negative dose-response relationship was observed: a greater number of chronic diseases was associated with poorer inhibitory control. Specifically, each additional chronic disease was associated with an average decrease of approximately 0.178 units in zBIS. This finding suggests that the link between chronic disease and impaired inhibitory control may reflect not only differences in disease thresholds, but also the cumulative burden of multiple conditions.

From a mechanistic standpoint, inhibitory control depends largely on the coordinated functioning of control systems, including the prefrontal–basal ganglia circuitry and the frontoparietal control network. The structural and functional states of these networks may also be affected by metabolic abnormalities and vascular risk factors (59, 60). Chronic diseases in older adults often present as clusters of comorbidities. For example, multiple cardiometabolic and vascular-related chronic conditions may coexist and interact, jointly affecting older adults' inhibitory control through intertwined metabolic and vascular mechanisms. At the metabolic level, chronic hyperglycemia, metabolic dysregulation, and impaired insulin signaling may disrupt brain energy metabolism and neuronal homeostasis, and are associated with susceptibility to neurodegenerative processes (61–63). Regarding vascular and inflammation-related pathways, cerebrovascular risks such as hypertension are associated with impaired white matter microstructure and decreased executive function. This also suggests that oxidative stress, inflammatory responses, and alterations in blood-brain barrier function may be involved (64). In this sense, chronic disease metrics can to some extent approximate an individual's cumulative burden of multiple diseases, and also provide a consistent explanatory framework for the dose-response trends observed.

### 4.3 Differences in the association between 24-h activity patterns and inhibitory control in older adults across chronic disease conditions

In the overall model, the interaction term between 24-h activity patterns and chronic disease status did not reach statistical significance, suggesting that the available data are insufficient to provide adequate statistical evidence to conclude that this association varies by chronic disease status. Therefore, the stratified proportional substitution analysis should be considered an exploratory analysis. Although the point estimates associated with MVPA appear numerically larger in the chronic disease group, this pattern should not be interpreted as evidence of a statistically significant difference between groups, particularly given the smaller sample size in the non-chronic disease group, which results in wider confidence intervals.

Prior research offers helpful context for this possible heterogeneity. For example, a meta-analysis pooling data from 14 multinational cohort studies by Jin et al. reported that a greater burden of cardiometabolic comorbidities was linked to poorer cognitive performance (65). The same work also suggested that unhealthy lifestyle factors, particularly low levels of physical activity, can strengthen this negative association, pointing to lifestyle as a plausible pathway connecting cardiometabolic multimorbidity with cognitive decline. Drawing on longitudinal CLHLS data, (72) found that multimorbidity was linked to an elevated risk of cognitive impairment, whereas healthier lifestyle profiles, including higher levels of physical activity, were associated with a reduced risk (66). Although these studies assessed broader cognitive outcomes, they suggest that lifestyle behaviors may be especially relevant when disease burden is higher. Taken together with earlier evidence and the present findings, a cautious interpretation is possible: in older adults with a heavier chronic disease burden, modifiable lifestyle behaviors, especially MVPA, may have a larger influence on inhibitory control performance. As a result, when time is reallocated from other behaviors toward MVPA, the link between MVPA and inhibitory control may be easier to detect. This interpretation is consistent with evidence in cardiometabolic conditions showing that exercise can improve Stroop-based inhibitory control performance (67). One plausible explanation is that chronic disease in later life often comes with metabolic disturbances, persistent low-grade inflammation, and reduced cardiopulmonary capacity (10, 68, 69). These changes may weaken the physiological foundation of inhibitory control, but they may also create greater potential for improvement. In this context, increases in MVPA may be more likely to produce measurable benefits in inhibitory control. This interpretation remains speculative and is based on observed patterns rather than direct causal evidence. Future studies should incorporate more detailed clinical information, such as disease type, severity, and level of control, and examine these hypotheses in larger samples using longitudinal or intervention-based designs.

However, evidence also indicates that the contribution of activity at different intensities may differ across populations. Using longitudinal data from older adults with chronic diseases, Volders et al. reported that for individuals who struggle to sustain sufficient MVPA over the long term, increasing LPA in real-life settings may be more feasible (70). That study further suggested that increases in LPA could be associated with larger improvements in inhibitory control than MVPA. This differs from our findings, in which LPA reallocations were not statistically significant. Such discrepancies may reflect differences in chronic disease type and severity, cultural and lifestyle contexts, and heterogeneity in physical activity measurement and statistical methods across studies.

## 5 Advantages and limitations

This study examined how the overall 24-h activity compositions and their redistribution relate to inhibitory control in older adults. Using accelerometer-based measures of daily activity, CoDA and proportional substitution approaches were applied while adjusting for multiple confounders. Chronic disease status was included within the same framework, and additional sensitivity analyses used the number of chronic conditions to represent disease burden, allowing the robustness of different chronic disease measures to be evaluated. This study has the following limitations. First, the sample sizes across subgroups were unevenly distributed, which may have compromised the statistical power required to test for effect modification by chronic diseases; therefore, statistically non-significant interaction results should be interpreted with caution, and the results of the stratified analysis should be considered exploratory findings. Second, although there is some overlap between the sample in this study and previously published literature, this study focuses on chronic diseases in older adults (including both those with and without chronic diseases), and both the research questions and analytical frameworks differ; these differences have been compared in detail in the Supplementary material. Third, the cross-sectional design limits the establishment of causal inferences. Fourth, wrist-worn accelerometers have inherent limitations in behavioral classification; the wrist-worn position and counting thresholds make it difficult to accurately distinguish between postures or activity contexts, particularly between sedentary behavior and low-intensity activity, and they cannot capture activities during periods when the device is not worn, such as swimming or bathing. Finally, information on chronic diseases was self-reported and lacked key details such as disease duration, severity, and control status; sensitivity analyses focusing solely on the number of chronic diseases are insufficient to fully reflect disease heterogeneity.

## 6 Conclusion

Among 24-h activity patterns, MVPA is the component most closely associated with inhibitory control performance among older adults. Increasing MVPA by proportionally reducing other activities correlates with improved inhibitory control performance. At the same time, chronic disease status was independently negatively associated with inhibitory control, with older adults with chronic diseases exhibiting lower overall inhibitory control. No statistically significant interaction was detected between 24-h activity patterns and chronic disease status. The stratified results are exploratory and require confirmation in larger, adequately powered studies. Given the limited sample size, this finding requires further validation in larger samples combined with longitudinal studies.

## Data Availability

The datasets presented in this article are not readily available because to protect the privacy of older adults, the data cannot be shared publicly. Requests to access the datasets should be directed to Lin Wang, wanglin123@126.com.
