# Supplementary material for: Association between 24-h activity patterns and inhibitory control in community-dwelling older adults: a cross-sectional study based on chronic disease status
Source: Front Public Health. 2026 Apr 16;14:1780172. doi: 10.3389/fpubh.2026.1780172 (PMC13128597; doi:10.3389/fpubh.2026.1780172)
Supplement: Supplementary file 1 [file Table_1.docx]

Supplementary Material

Supplementary Table 1. STROBE checklist for cross-sectional observational studies

| **Item No.** | **Section** | **Recommendation** | **Status** | **Location in revised manuscript** |
| --- | --- | --- | --- | --- |
| 1a | Title and abstract | State the study design in the title or abstract using a common term. | Reported | Title; Abstract – Method ('A cross-sectional design was employed…') |
| 1b | Title and abstract | Provide an informative and balanced abstract of what was done and found. | Reported | Abstract – Objective, Method, Results, Conclusion |
| 2 | Background/rationale | Explain the scientific background and rationale. | Reported | Introduction – paragraphs 1–4 |
| 3 | Objectives | State specific objectives and any prespecified hypotheses. | Reported | Introduction – final paragraph |
| 4 | Study design | Present key elements of study design early in the paper. | Reported | Abstract – Method; Materials and methods – Participants |
| 5 | Setting | Describe setting, locations, and relevant dates of recruitment/data collection. | Reported | Materials and methods – Participants |
| 6a | Participants | Give eligibility criteria and sources/methods of participant selection. | Reported | Materials and methods – Participants |
| 6b | Participants | For matched studies, give matching criteria and numbers of exposed/unexposed. | Not applicable | This cross-sectional study did not use a matched design. |
| 7 | Variables | Clearly define outcomes, exposures, predictors, confounders, and effect modifiers. | Reported | Measures; Covariates; Statistical analysis |
| 8 | Data sources/measurement | For each variable, give data sources and measurement details; describe comparability across groups if relevant. | Reported | Assessment of 24-hour activity patterns; Assessment of inhibitory control; Assessment of chronic disease; Covariates |
| 9 | Bias | Describe efforts to address potential sources of bias. | Reported | Assessment of 24-hour activity patterns (compliance checks, logs); Statistical analysis; Advantages and Limitations |
| 10 | Study size | Explain how study size was determined. | Reported | Participants (a priori calculation for primary regression model) |
| 11 | Quantitative variables | Explain handling of quantitative variables and any groupings chosen. | Reported | Assessment of chronic disease; Covariates; Statistical analysis |
| 12a | Statistical methods | Describe all statistical methods, including confounder control. | Reported | Statistical analysis |
| 12b | Statistical methods | Describe methods used for subgroup and interaction analyses. | Reported | Statistical analysis (Model 2, stratified proportional substitution analyses, sensitivity analysis) |
| 12c | Statistical methods | Explain how missing data were addressed. | Reported | Participants (exclusions for insufficient wear time, incomplete baseline information, or missing Stroop data); Supplemental Figure 1 |
| 12d | Statistical methods | If applicable, describe analytical methods accounting for sampling strategy. | Not applicable | Convenience sampling is stated in Participants; no complex sampling design was used |
| 12e | Statistical methods | Describe any sensitivity analyses. | Reported | Statistical analysis; Results – Sensitivity Analysis; Supplementary Table 5 |
| 13a | Participants | Report numbers of individuals at each stage of the study. | Reported | Participants; Results – Participant characteristics; Supplemental Figure 1 |
| 13b | Participants | Give reasons for non-participation or exclusion at each stage. | Reported | Participants; Supplemental Figure 1 |
| 13c | Participants | Consider use of a flow diagram. | Reported | Supplemental Figure 1 |
| 14a | Descriptive data | Give characteristics of participants and information on exposures/confounders. | Reported | Results – Participant characteristics; Table 1 |
| 14b | Descriptive data | Indicate number of participants with missing data for each variable of interest. | Reported | Participants and Supplementary Figure 1. Reasons for exclusion and numbers excluded are reported, and the final analytic sample included participants with valid accelerometer data, Stroop task results, and complete baseline information. |
| 15 | Outcome data | Report outcome events or summary measures. | Reported | Results; Tables 1–4 |
| 16a | Main results | Give unadjusted and, if applicable, adjusted estimates and their precision; state adjusted confounders. | Reported | Results; Tables 2–4; Supplementary Tables 2–5 |
| 16b | Main results | Report category boundaries when continuous variables were categorized. | Reported | Assessment of chronic disease; Covariates; Notes to Supplementary Table 5 |
| 16c | Main results | If relevant, translate estimates into absolute risk for a meaningful time period. | Not applicable | The outcome (zBIS) was a continuous standardized score rather than a risk measure. |
| 17 | Other analyses | Report subgroup analyses, interactions, and sensitivity analyses. | Reported | Results – interaction model, stratified analyses, and sensitivity analysis; Figure 1; Supplementary Table 5 |
| 18 | Key results | Summarize key results with reference to study objectives. | Reported | Discussion – opening sections; Conclusion |
| 19 | Limitations | Discuss limitations, including potential bias or imprecision. | Reported | Advantages and Limitations |
| 20 | Interpretation | Provide a cautious overall interpretation considering objectives, limitations, multiplicity, and evidence. | Reported | Discussion; Conclusion |
| 21 | Generalisability | Discuss generalisability (external validity) of the results. | Reported | Discussion and Conclusion (community-dwelling older adults are specified) |
| 22 | Funding | Give the source of funding and the role of the funders. | Reported | Supported by three funding sources |

Notes: zBIS, standardized Balanced Integration Score; ilr, isometric log-ratio; BMI, body mass index; SB, sedentary behavior; LPA, light-intensity physical activity; MVPA, moderate-to-vigorous-intensity physical activity.

Supplemental Figure 1. Flow diagram

First wave participants: n = 112

Recruitment sites: East and West residential communities, Mafangshan Campus, Wuhan University of Technology

Recruitment period: September–November 2024

Total number of participants included in the final analysis

n=121

Included in this wave: n = 16

Excluded: n = 10

Insufficient GT3X wear time n = 6 Incomplete baseline information n = 1 Missing Stroop task test data n = 3

Excluded: n = 7

Insufficient GT3X wear time n = 5 Missing Stroop task test data n = 2

Second wave participants: n = 26

Recruitment site: Youli community, Wuhan University of Technology

Recruitment period: March–April 2025

Included in this wave: n = 105

Supplementary Table 2. Comparison of study design, research aims, and analytical strategies between the present study and Wang et al. (2026)

| **Project** | **The Frontiers article** | **This study** |
| --- | --- | --- |
| Data source | Derived from the same parent community-based project; prior publication from the same study wave. | Distinct analysis derived from the same parent community-based project; partial participant overlap with the Frontiers article. |
| Analytic sample and participant overlap | N = 75; restricted to older adults with chronic diseases. | N = 121; includes 86 participants with chronic diseases and 35 without chronic diseases; 75 participants overlapped with the Frontiers article (75/121, 61.98%). |
| Study population | Older adults with chronic diseases. | Community-dwelling older adults with and without chronic diseases. |
| Recruitment sites | the East and West residential communities of Wuhan University of Technology | the East and West residential communities on the Mafangshan Campus and the Youli community，Wuhan University of Technology |
| Recruitment dates | Sep–Nov 2024 | September 2024 to April 2025 |
| Inclusion criteria | 1) age ≥60 years; (2) ability to independently complete computer-based testing tasks; (3) presence of at least one chronic disease. | (1) age ≥60 years; (2) community-dwelling; (3) able to ambulate independently and perform usual activities of daily living without assistance; (4) able to communicate and understand task instructions and to complete the Stroop practice trials; and (5) willing to participate and provide written informed consent. |
| Exclusion criteria | (1) severe cognitive impairment (e.g., professionally diagnosed dementia); (2) hearing, visual, or communication impairments; (3) missing 24-h activity data. | (1) self-reported physician diagnosis of dementia or other major neurological disease; (2)hearing, vision, or communication impairments that precluded completion of the Stroop task; (3) severe health conditions that made unsupervised physical activity medically unsafe or substantially limited functional mobility. |
| Primary research question | Examined how 24-h activity patterns were associated with executive function among older adults with chronic diseases. | Examined the association between 24-h activity patterns and inhibitory control in a broader community sample, and further tested the independent association and potential modifying role of chronic disease status/burden. |
| Primary endpoint | Broader executive function | Specifically inhibitory control |
| Outcome definition | Executive function, including inhibitory control: the dependent variable was the correct reaction time for incongruent Stroop trials; task outcomes were also converted to task-standardized z-scores to enable comparisons across tasks. | Inhibitory control: the Stroop task recorded AC and RT; zBIS = z(AC) − z(RT). zBIS was computed separately for the congruent and incongruent conditions, and the final Stroop-based score was calculated as zBIS(incongruent) − zBIS(congruent). Higher zBIS values indicate better inhibitory control. |
| Role of chronic disease variable | Chronic disease status served as a sample restriction criterion. | Chronic disease status was analyzed as an independent exposure, as a potential effect modifier, and in sensitivity analyses as disease burden (number of chronic conditions). |
| Accelerometer validity | ActiGraph wGT3X-BT; worn on the non-dominant wrist; 7 consecutive days; epoch = 60 s; valid day ≥ 10 h; ≥ 3 valid days (2 weekdays + 1 weekend day); removed only for bathing/swimming, etc.; paper-based activity/sleep logs were recorded for calibration. | ActiGraph wGT3X-BT; worn on the non-dominant wrist for 7 consecutive days (5 weekdays + 2 weekend days), removed only for bathing/swimming, etc.; epoch = 60 s; valid day ≥ 10 h; ≥ 3 valid days (2 weekdays + 1 weekend day); paper logs recorded bedtime and wake time for calibration. |
| Reallocation scheme | One-to-one isotemporal substitution | One-to-three proportional reallocation |
| Covariates in models | Age, gender, education level | Model 1：age, gender, BMI, education level, and economic status, chronic disease status；Model 2 included an interaction term between the activity patterns and chronic disease status. In the sensitivity analysis, chronic disease status was replaced with the number of chronic diseases. |
| Key added value | Provided evidence within a chronic-disease-only sample using one-to-one isotemporal substitution. | Extends prior work by adding a disease-free comparison group, estimating the adjusted main effect of chronic disease status, exploring potential effect modification by chronic disease status, applying one-to-three proportional reallocation with bootstrap CIs, and evaluating chronic disease burden in sensitivity analysis. |

Supplementary Table 3. Compositional linear regression analysis

| variable | Model 1 | | | Model 2 | | | Model 3 | | |
| --- | --- | --- | --- | --- | --- | --- | --- | --- | --- |
|  | *β* (95%CI) | *P* | Adjusted *R^2^* | *β* (95%CI) | *P* | Adjusted *R^2^* | *β* (95%CI) | *P* | Adjusted *R^2^* |
| Sleep | -0.45(-1.09,0.19) | 0.17 | 0.21 | -0.37 (-1.01,0.28) | 0.26 | 0.230 | -0.55(-1.34,0.23) | 0.16 | 0.273 |
| SB | -1.02(-1.57,-0.48) | 0.0003 |  | -1.02 (-1.57,-0.48) | 0.0003 |  | -0.77 (-1.36,-0.17) | 0.013 |  |
| LPA | -0.08(-0.64,0.49) | 0.79 |  | -0.13 (-0.69,0.44) | 0.66 |  | -0.10(-0.67,0.47) | 0.73 |  |
| MVPA | 0.67(0.28,1.06) | 0.0009 |  | 0.72 (0.33,1.11) | 0.0004 |  | 0.55(0.16,0.94) | 0.007 |  |

Note: Model 1 is unadjusted; Model 2 is adjusted for sex and age only; Model 3 is additionally adjusted for BMI, education, socioeconomic status, and chronic disease status based on Model 2.

Supplementary Table 4. Full adjusted regression coefficients for Model 1

| **Predictor** | **Estimate** | **SE** | **t value** | **p value** |
| --- | --- | --- | --- | --- |
| ilr.comp1 | -0.16291 | 0.33223 | -0.49 | 0.62486 |
| ilr.comp2 | 0.09543 | 0.24606 | 0.388 | 0.6989 |
| ilr.comp3 | 0.52827 | 0.19496 | 2.71 | 0.00782 |
| Sex 1 | 0.32535 | 0.16749 | 1.942 | 0.05464 |
| Age | 0.01779 | 0.01165 | 1.527 | 0.12966 |
| BMI | -0.038 | 0.03119 | -1.218 | 0.22573 |
| Education level 1 | 0.44843 | 0.28751 | 1.56 | 0.1217 |
| Education level 2 | 0.5151 | 0.31836 | 1.618 | 0.10853 |
| Socioeconomic status 1 | 0.05311 | 0.15482 | 0.343 | 0.73221 |
| Chronic disease status 1 | -0.43362 | 0.17775 | -2.439 | 0.01631 |

Supplementary Table 5. Sensitivity analysis

|  | *β* | *SE* | *t* | *p* |
| --- | --- | --- | --- | --- |
| (Intercept) | -1.580 | 1.202 | -1.315 | 0.191 |
| **ilr1 (sleep vs SB/LPA/MVPA)** | -0.087 | 0.325 | -0.268 | 0.789 |
| **ilr2 (SB vs LPA/MVPA)** | 0.091 | 0.239 | 0.382 | 0.704 |
| **ilr3 (LPA vs MVPA)** | 0.485 | 0.191 | 2.534 | 0.013 |
| Sex (level 1 vs 0) | 0.347 | 0.164 | 2.118 | 0.036 |
| Age (years) | 0.019 | 0.011 | 1.647 | 0.102 |
| BMI | -0.031 | 0.031 | -1.013 | 0.313 |
| Education (level 1 vs 0) | 0.637 | 0.287 | 2.218 | 0.029 |
| Education (level 2 vs 0) | 0.695 | 0.314 | 2.216 | 0.029 |
| ses (level 1 vs 0) | 0.053 | 0.151 | 0.353 | 0.725 |
| Number of chronic diseases | **-0.178** | 0.052 | -3.397 | **0.0009** |

Note: compositional linear regression results for zBIS with chronic disease burden quantified as the number of chronic diseases , replacing binary chronic disease status;

The 24-hour activity patterns was ordered as Sleep, SB, LPA, and MVPA and transformed using pivot isometric log-ratio coordinates (default pivot = Sleep);

For number of chronic diseases, the 95% CI was −0.28 to −0.07;

Categorical variables were coded as follows: sex (0 = male, 1 = female), Education (Ed0 = primary school or below, Ed1 = secondary school, Ed2 = college or above), and ses (ses0 ≤5000 RMB, ses1 >5000 RMB).
